# Sibling Configuration Predicts Individual and Descendant Socioeconomic Success in a Modern Post-Industrial Society

**DOI:** 10.1371/journal.pone.0073698

**Published:** 2013-09-06

**Authors:** David W. Lawson, Arijeta Makoli, Anna Goodman

**Affiliations:** 1 Department of Anthropology, University College London, London, United Kingdom; 2 Centre for Health Equity Studies (CHESS), Stockholm University/Karolinska Institute, Stockholm, Sweden; 3 Faculty of Epidemiology and Population Health, London School of Hygiene & Tropical Medicine, London, United Kingdom; Durham University, United Kingdom

## Abstract

Growing up with many siblings, at least in the context of modern post-industrial low fertility, low mortality societies, is predictive of relatively poor performance on school tests in childhood, lower levels of educational attainment, and lower income throughout adulthood. Recent studies further indicate these relationships hold across generations, so that the descendants of those who grow up with many siblings are also at an apparent socioeconomic disadvantage. In this paper we add to this literature by considering whether such relationships interact with the sex and relative age of siblings. To do this we utilise a unique Swedish multigenerational birth cohort study that provides sibling configuration data on over 10,000 individuals born in 1915–1929, plus all their direct genetic descendants to the present day. Adjusting for parental and birth characteristics, we find that the ‘socioeconomic cost’ of growing up in a large family is independent of both the sex of siblings and the sex of the individual. However, growing up with several older as opposed to several younger siblings is predictive of relatively poor performance on school tests and a lower likelihood of progression to tertiary education. This later-born disadvantage also holds across generations, with the children of those with many older siblings achieving lower levels of educational attainment. Despite these differences, we find that while individual and descendant income is negatively related to the number of siblings, it is not influenced by the relative age of siblings. Thus, our findings imply that the educational disadvantage of later-born children, demonstrated here and in numerous other studies, does not necessarily translate into reduced earnings in adulthood. We discuss potential explanations for this pattern of results, and consider some important directions for future research into sibling configuration and wellbeing in modern societies.

## Introduction

Children in relatively large families, at least in the context of ‘modern’ post-industrial low fertility, low mortality populations, are now well established to be at an increased risk of poor outcomes across multiple dimensions of wellbeing. For example, children in larger families are known to perform relatively poorly on IQ tests and formal educational assessments [1], [2], [3], [4], [5] and also show signs of poorer physical health [6], [7], [8], [9]. There is also good reason to believe that these associations are causal, as in most studies estimated relationships have proven robust to statistical adjustment for parental characteristics (but see [10], [11]). Several studies also confirm that children in large families are disadvantaged in terms of both maternal and paternal time-allocation [1], [12], [13], [14], [15]. Furthermore, reflecting the need to feed, clothe, and house more children, parents in large family households also report increased levels of economic hardship, even after adjustment for ethnicity, socio-economic position and other factors [16], [17]. These findings are consistent with simple theoretical models of household resource dilution, which posit that, all else being equal, individuals raised in larger families are disadvantaged because the presence of siblings dictates a division of finite parental resources [4]. These findings are also supportive of parallel theoretical frameworks in both economics and evolutionary biology that posit a parental investment trade-off between offspring quantity and ‘quality’ [18], [19].

In a recent study [20], we further demonstrated that negative relationships between family size and child outcomes extend into adulthood and across generations, meaning that variation in fertility may have long reaching consequences for social and health inequalities. Utilizing a unique multigenerational Swedish cohort, we demonstrated that children, grandchildren and even great-grandchildren of high fertility individuals appear to suffer negative socioeconomic consequences in terms of schoolmarks, educational attainment and adult income [20]. These intergenerational effects remained significant after the adjustment for the family sizes of intervening generations. This suggests that the socioeconomic costs of large family size are caused by the dilution of inherited wealth (see also [21], [22]) and other forms of parental investment, rather than covariation in the fertility of parents and children.

A less certain issue is the extent to which the socioeconomic effects of large family size may be influenced by the characteristics of siblings themselves, including their relative age and sibling sex. In ‘traditional’ agrarian pre-demographic transition populations (i.e. high fertility, high mortality), a common finding is that number of brothers, but not sisters, has strong negative effects on adult socioeconomic outcomes among males (e.g. [23], [24], [25]). This reflects the fact that sons are often in direct resource competition for parental resource transfers required for marriage and the establishment of new households. Following systems of primogeniture, inheritance practices also frequently, but not always (e.g. [26]), favour children born earlier in the birth order [27]. Furthermore, there is evidence from anthropological and demographic studies that sons and earlier-born children are often treated preferentially by parents during childhood (e.g. [28], [29]), although such biases are not cross-culturally universal in traditional societies (e.g. [30]). The ultimate origins of differential treatment of children by sex and birth order remain the subject of debate by anthropologists (see [23], [27], [31]).

Do parents in ‘modern’ post-industrial low fertility, low mortality societies also show patterns of biased investment? With regard to sex-biased parental investment, a number of studies have suggested that several modern European and North American populations are characterized by some degree of son preference [32]. For example, studies of the United States population have shown that parents with more sons than daughters are more likely to marry and less likely to divorce [33], [34] and that, on average, fathers’ earnings increase to a larger extent in the years following the birth of a son relative to the birth of a daughter [35]. In a longitudinal study spanning the first 10 years of life in British children, Lawson and Mace [12] found mothers spent slightly more time on average engaging in active childcare activities with daughters. Fathers, however, showed stronger signs of bias in the opposite direction, spending more time with sons, particularly after infancy. On the other hand, Sweden and several other Scandinavian countries show indications of a slight daughter preference, as indirectly evidenced by higher rates of having a third child in two-son families than in two-daughter families [36]. To the extent that parents do invest preferentially in children of a particular sex, one might expect siblings of that sex to confer larger detrimental effects. To date the evidence that brothers vs. sisters have different effects on child educational outcomes is very mixed [5], [37]. For example, studies of the United States population have concluded that brothers rather than sisters are associated with lower educational attainment (e.g. [38]), while others studies suggest the opposite pattern (e.g. [39]) and others still conclude there is no effect of sibling sex (e.g. [40]). We are not aware of studies that have examined sibling sex effects on alternative indicators of adult socioeconomic position and on the socioeconomic outcomes of subsequent generations.

With regard to birth order, a number of time-allocation studies have suggested that later-born children receive less parental investment in modern populations [12], [41]. An important conclusion from this literature is that such a later-born deficit may not be the product of conscious strategic effort of parents. Rather it may occur as the by-product of equalised treatment of children at different ages within the family and the fact that older siblings monopolize parental attention before the birth of later-born children [4], [41], [42]. Furthermore, sibling relationships have also been suggested to be more beneficial to early-born children. This may be because the act of teaching younger siblings promotes cognitive development (e.g. [43]). Proponents of ‘confluence theory’ also argue that relative sibling age influences cognitive development, not because it reduces shares of finite parent investment, but rather because it alters the intellectual climate of the family unit – assumed to be a function of the average age of all household members. Early born children are therefore seen to be at an overall advantage because they experience more time in households with less children and thus a relatively sophisticated intellectual family environment [44].

In line with this literature, many studies have reported that later-born children perform significantly worse on educational and IQ tests [2], [3], [5], [45], [46]. However, fewer studies directly compare the consequences of having younger vs. older siblings, and relatively little is known about whether such effects also influence adult socioeconomic position and the transmission of socioeconomic resources across generations. This is important because although there are strong effects of birth order on educational attainment, inheritance sums may be bequeathed equally to all children in modern populations, potentially offsetting the disadvantage of late birth order. Furthermore, there are indications that later-born children may be advantaged in other meaningful ways that could offset their early disadvantage in childhood education. For example, Lawson and Mace [47] found that British children with many older vs. younger siblings scored significantly better on measures of child mental health, and may therefore be less likely to suffer a range of adverse outcomes as adults [48].

In this paper, we build on the analyses in Goodman et al. [20] to contribute new data on the effects of sibling relative age and sibling sex upon own and descendant socioeconomic outcomes. Utilizing a unique multigenerational Swedish cohort of 14,000 children and their biological descendants, and adjusting for a number of important covariates, we 1) compare the relationship of number of brothers versus number of sisters to childhood and adult measures of socioeconomic position; 2) compare the relationship of number of older siblings versus number of younger siblings to these same outcomes; and 3) examine how far any of these relationships are also observed with respect to socioeconomic outcomes in children and grandchildren of the cohort member.

## Methods

### Ethics Statement

All data were derived from the Uppsala Multigenerational Birth Cohort Study (UBCoS), a unique Swedish dataset that tracks over 14 000 individuals born in the early 1900s and all their descendants to the present day. UBCoS was approved by the Regional Ethics committee in Stockholm (dnr 03–117, dnr 04–944T and dnr 2009/1115–32).

### Sample Selection

Our sample comprises all live births at the Uppsala University Hospital between 1915 and 1929. This hospital delivered an estimated 75% of births in Uppsala city and 50% of births in surrounding rural parishes. This birth cohort is nationally representative of Sweden in terms of infant mortality and fertility [49], albeit with a somewhat higher proportion of infants from urban areas (46% vs. 31% nationally [20]).

For our analyses of sibling sex, we used the full birth cohort of 14,192 infants as our starting point, and excluded those who systematically lacked data on child and/or adult socioeconomic outcomes. This included cohort members who were never traced (N = 167), who died (N = 2047) or permanently emigrated before 1970 (N = 110). We also excluded those who were missing reliable data on number of brothers or sisters (N = 929). This remaining study population of 10,939 represents 85% of those who survived to age 10, i.e. who survived long enough to receive any outcome measure of interest to this study. For our analyses of sibling age, we further restricted our analyses to the 7091 study members born 1915–1924 (91% of those who survived to age 10). We did this in order in increase our ability to measure accurately the child’s number of younger siblings, as sibling data were last collected in 1930).

We supplemented this data by linking cohort members to all biological descendants born up to 31^st^ December 2009, using the Swedish Multigenerational Register (estimated completeness 97.7% for paternity, 99.6% for maternity [49]). Our analyses focus on outcomes in the children and grandchildren of cohort members; as judged by the distribution of birth years, these generations were essentially complete by 2009 [20].

### Sibling Configuration

Data on the number of brothers and sisters, and older and younger siblings was available from multiple sources. First, obstetric records were available for all cohort members, and provided two sources of data: (a) the mother’s previous number of live births was recorded in the obstetric record of each cohort member, irrespective of whether those previous births were at Uppsala Hospital or not. From 1924 this was done separately for previous male and previous female births; and (b) we could identify older and younger siblings born to the same woman at Uppsala Hospital across the period of data collection (1915–1929). Second, census data were available for the 68% of cohort members successfully traced to the 1930 Swedish census. This recorded information on the sex and birth year of all household members, including text descriptions that allowed us to identify their relationship to the index child (see File S1).

We triangulated data from across these data sources to identify the number of older and younger siblings for each cohort member, and their number of brothers and sisters. This involved comparing the number of siblings of each type reported in each data source, and using the highest number (see File S1 for more detail). Pearson correlations between the number of siblings identified in the different data sources ranged from 0.70 (for no. brothers) to 0.86 (for no. older siblings). Note that we did not count twins or triplets as either older or younger siblings, but used obstetric information to create a separate variable to capture twin/triplet status.

In estimating a child’s number of siblings, we included not only full siblings (95% of all siblings in the 1930 census) but also foster/adoptive siblings (1% in the 1930 census) and step- or half-siblings (4% in the 1930 census). We did this to capture the sibship experienced while growing up and also because we were unable to distinguish between full siblings and maternal half siblings in the obstetric data. Among those with census data, our findings were unchanged in sensitivity analyses which only counted full siblings and/or which were restricted to the 93% of cohort members with no adoptive, step- or half-siblings. Our findings were also unchanged in sensitivity analyses restricted to cohort members born 1915–1919 who, judging by the age distribution of their siblings, had by 1930 acquired the majority of their younger siblings that would ever be born (see File S1). The gender distribution of older siblings suggested that we had captured older brothers and sisters equally (see File S1).

### Indicators of ‘Socioeconomic Success’

We used archive and register data to assign three indicators of socioeconomic success:

- Schoolmarks: standardised average marks across all compulsory subjects in elementary school (collected age 10 in the cohort members, age 16 in their grandchildren: not available for the intervening generation; see also [50]).
- Entering university: ever entering university or equivalent, if aged 21 or over. Education data was available in 1960, 1970, and then yearly from 1985–2008.
- Family income: disposable family income, standardised each calendar year by age and sex and then averaged across all available calendar years in which the descendant was aged 21–65. Income data was available in 1970, and then yearly from 1990–2008.

Each indicator of socioeconomic success was used as an individual-level outcome for the cohort members themselves. For the children and grandchildren of the cohort members, we generated averages across all available descendants e.g. proportion of children entering university, average income among grandchildren. Goodman et al. [20] and the supplementary information therein provide full details on how each of these measures were derived.

### Parental Socioeconomic Position and Other Early-life Characteristics

We measured parental socioeconomic position and other early-life characteristics using the archived obstetric records. These records provided data on cohort members’ birthweight and gestational age, on the mother’s age and marital status, and on the occupational social class of the head of the household (see Table 1). Social class was coded using the Swedish socio-economic classification scheme [51] and was taken from the obstetric records of the cohort member if available (92%), or from the records of their siblings, from the school archive or from the 1930 census if missing (taking the record closest in time). We included these as covariates as we have previously shown that all independently predict the educational outcomes of the children and/or grandchildren of the cohort members [50]. Our results were unchanged in sensitivity analyses, which excluded birthweight as a covariate, as this typically increases with mother’s parity and so could conceivably mediate rather than confound associations with number of older versus younger siblings.

**Table 1:** Characteristics of Uppsala Multigenerational Birth Cohort Members.

	Variable	Categories	N (%) cohort members in study population used for analyses of….	
			Sibling sex (N = 10,939)	Sibling age (N = 7109)
Early-life	Sex	Male	5628 (52%)	3666 (52%)
characteristics		Female	5311 (49%)	3443 (48%)
	Birth year	1915–1917	1460 (13%)	1810 (25%)
		1918–1920	1747 (16%)	2040 (29%)
		1921–1923	2128 (20%)	2414 (34%)
		1924–1926	2657 (24%)	845 (12%)
		1927–1929	2947 (27%)	–
	Low birthweight	No	10,386 (96%)	6784 (96%)
	(<2500 g)	Yes	477 (4%)	283 (4%)
	Preterm birth (<37	No	9825 (93%)	6393 (93%)
	weeks gestation)	Yes	762 (7%)	479 (7%)
	Child a twin/triplet	No	10,651 (97%)	6936 (98%)
		Yes	288 (3%)	173 (2%)
	Mother’s age at	<19 years	666 (6%)	399 (6%)
	child’s birth	20–24 years	2998 (27%)	1870 (26%)
		25–29 years	3081 (28%)	2015 (28%)
		30–34 years	2188 (20%)	1428 (20%)
		≥35 years	2006 (18%)	1395 (20%)
	Mother unmarried	No	8738 (80%)	5691 (80%)
	at child’s birth	Yes	2183 (20%)	1394 (20%)
	Parental	High/med non-manual	914 (9%)	592 (9%)
	social class	Low non-manual	751 (7%)	547 (8%)
	near birth	Farmer/self-employed	2076 (19%)	1270 (18%)
		Skilled manual	1610 (15%)	1045 (15%)
		Unskilled manual	5394 (50%)	3498 (50%)
Sibling	Child’s no.	0	4716 (43%)	
characteristics	brothers	1	2842 (26%)	
		2	1609 (15%)	
		3+	1772 (16%)	
	Child’s no.	0	5075 (46%)	
	sisters	1	2830 (26%)	
		2	1620 (15%)	
		3+	1414 (13%)	
	Child’s no.	0		2749 (39%)
	older siblings	1		1758 (25%)
		2		967 (14%)
		3+		1635 (23%)
	Child’s no.	0		3800 (53%)
	younger siblings	1		1722 (24%)
		2		845 (12%)
		3+		742 (10%)

Numbers add to less than the total sample size for some characteristics because of missing data.

### Statistical Analyses

We used multivariable regression to investigate how the numbers of different types of sibling were associated with the socioeconomic success of cohort members and with the average success of their descendants. To facilitate comparisons of effect sizes across generations and across outcomes, we standardised all outcomes for each generation and used these in linear regression analysis. The only exception was for one of our binary outcomes (cohort member entering university), for which we used logistic regression and converted the log-odds to effect sizes [52].

We compared the effects of number of brothers versus sisters by entering both simultaneously as linear terms in a multivariable model, and then calculating the significance of the difference between the two coefficients. We did the same when comparing number of older versus younger siblings, except when the outcome was schoolmarks. In this last analysis we instead made the comparison between the coefficients using a piecewise approach, as there was evidence that the association between number of older siblings and schoolmarks was non-linear (p<0.001, all other p>0.05 for linearity). We adjusted all these analyses for the cohort member’s birthweight, gestational age, twin/triplet status, mother’s age, mother’s marital status, parental socio-economic position and birth year (categorised as in Table 1, all correlation coefficients ≤0.43 between early-life characteristics). We calculated confidence intervals using robust standard errors clustered by mother.

The frequency of missing data ranged from 0–3.4% for early life characteristics and from 2.7–11.8% for outcome characteristics (NB this excludes impossible outcomes, e.g. average descendant characteristics among a childless cohort member). All analyses handled missing data under an assumption of missing at random, using multiple imputation by chained equations [53] in Stata (5 imputations, including in our imputation model all variables and structure included in substantive models).

## Results

As shown in Table 1, the 10,939 cohort members used in our analysis of sibling sex had roughly equal numbers of brothers and sisters (mean 1.2 vs. 1.1). By contrast, the 7109 cohort members used in our analysis of sibling age had a larger number of older siblings than younger siblings (mean 1.7 vs. 0.9). This difference is largely due to the fact that sibling information was recorded for the last time in 1930 (when the original cohort members were aged 5–15 years old) and any younger siblings born after 1930 are therefore missing from our analysis. As for our three socio-economic outcomes, correlations between these were always positive but were not large (0.08≤r≤0.29 for all outcomes across all three generations, except for r = 0.56 between schoolmarks and tertiary education in the grandchild generation).

### Overall Estimated Effects of Family Size on Education and Income

**Figure 1:**
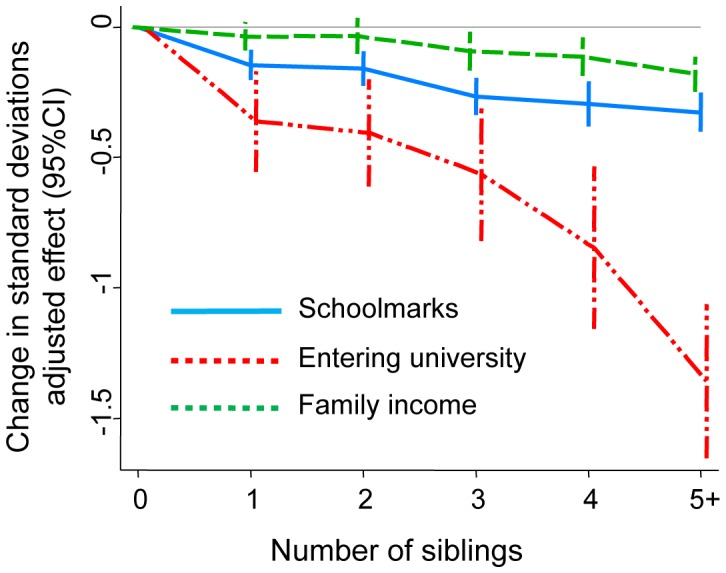
Association of the cohort members’ total family size with their schoolmarks educational attainment and adult income. Analyses based on the 10,939 individuals with valid data on sibling sex. Adjusted effects show the difference in standard deviations relative to zero siblings, based on multivariable models adjusting for the early-life characteristics shown in Table 1.

As previously stated elsewhere [20], total family size showed a strong association with both education and income in our cohort members (Figure 1). These effects were seen across the whole range of increasing family size, with a particularly strong association with the probability of entering university. For example, under 5% of those with five or more siblings entered university as opposed to 16% of those with no siblings, corresponding to an adjusted effect size of −1.36 standard deviations (95% CI −1.65, 1.06: see Figure 1).

### Sibling Sex, Education and Income

**Figure 2:**
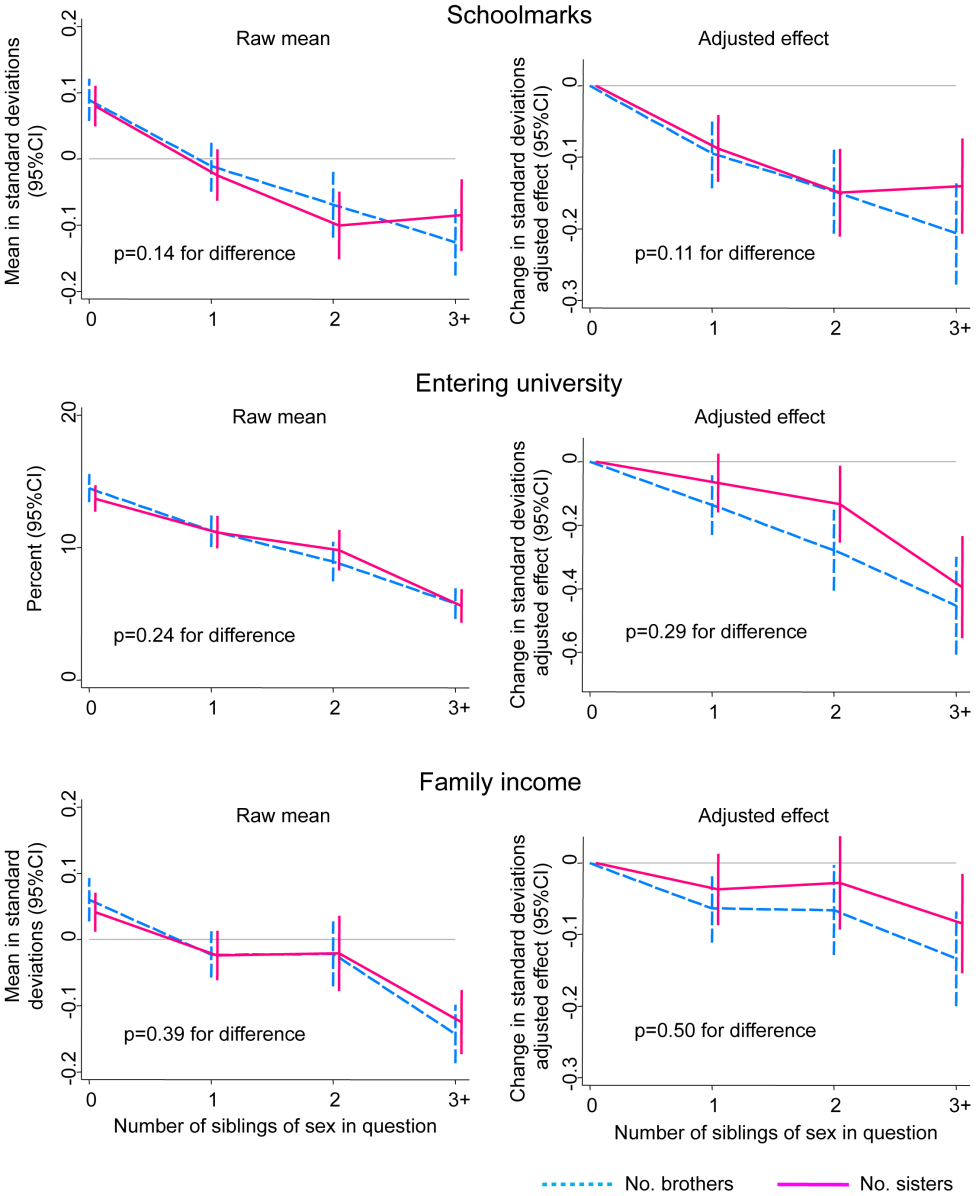
Association of the cohort members’ number of brothers versus sisters with the cohort members’ schoolmarks, educational attainment and adult income. p-values presented are for the difference in the effect of number of brothers versus sisters. In the left-hand column these p-values come from models only adjusting for these two sibling variables. In the right-hand column the p-values come from multivariable models additionally adjusting for the early-life characteristics shown in Table 1.

As presented in Figure 2, there was never evidence that the detrimental effects of having more siblings differed according to whether those siblings were brothers or sisters. Instead more siblings of either sex predicted progressively poorer socio-economic outcomes among cohort members, with particularly large effects upon the two educational outcomes. There was likewise no evidence that the sex of the siblings was important when we compared the effects of older bothers versus older sisters, and of younger brothers versus younger sisters, among the 6180 cohort members who had valid information of both sibling age and sex (all p>0.05 for difference). Finally, there was never evidence either for this or for subsequent analyses that the effects presented differed between males and female cohort members (all p>0.08 for interaction with sex of the cohort member, most p>0.2). For this reason Figure 2 and subsequent figures combine male and female cohort members.

### Sibling Relative Age, Education and Income

**Figure 3:**
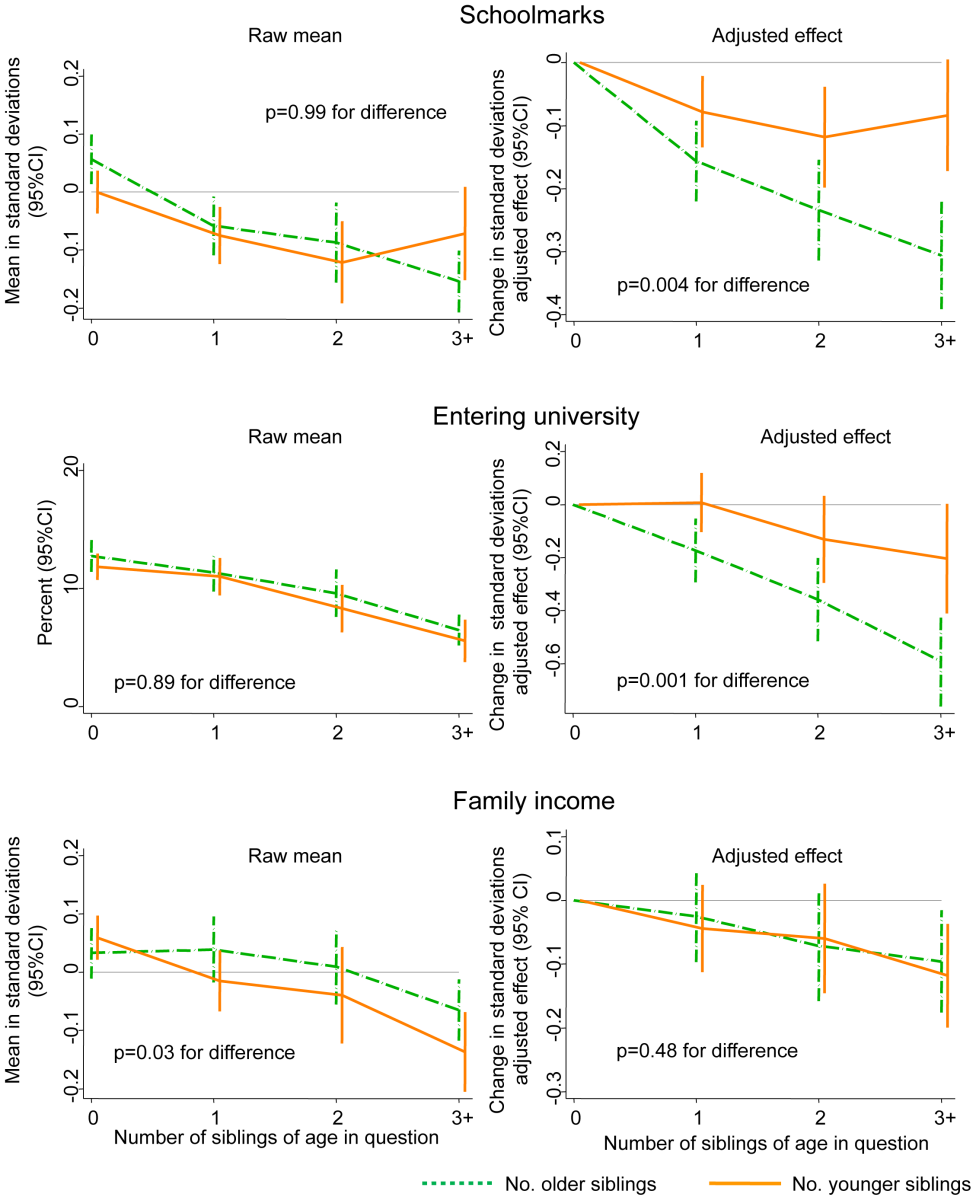
Association of the cohort members’ number of older versus younger siblings with the cohort members’ schoolmarks, educational attainment and adult income. p-values presented are for the difference in the effect of number of older versus younger siblings. In the left-hand column these p-values come from models only adjusting for these two sibling variables. In the right-hand column the p-values come from multivariable models additionally adjusting for the early-life characteristics shown in Table 1.

As presented in Figure 3, there was little or no evidence that the relative age of siblings affected our outcomes in unadjusted analyses. In adjusted analyses, by contrast, strong evidence emerged that the negative impact of older siblings was greater than that of younger siblings for the two educational outcomes. Adjusting one by one for the characteristics in Table 1 indicated that the difference between the unadjusted and the adjusted effects was largely driven by adjusting for whether the mother had ever been married. The negative confounding of this variable reflected the fact that early-born children were more likely than later-born children to be born to never-married mothers (e.g. 36% of first born children versus 16% of second-born and <5% thereafter). Being born to a never-married mother in turn predicted substantially poorer educational outcomes (e.g. 5% of the children of never-married mothers entered university, as opposed to 12% for ever-married mothers; see also [50]). By contrast, no effects of sibling age were observed upon income in adulthood after adjustment for other early-life characteristics.

These substantive findings were all almost identical in analyses restricted to those born 1915–1919, for whom most younger siblings that would ever be born had been born by 1930. Indeed for schoolmarks there was if anything an even greater difference between older and younger siblings effects after restricting to those born 1915–1919 (see File S1). As such, it did not seem that the observed differential effect of sibling age could be explained by greater measurement error with respect to younger siblings.

### Differential Effects of Sibling Configuration Across Generations

No intergenerational effects were seen for those variables which showed no effect on the cohort members themselves. Specifically, there was never evidence of a differential effect of number of brothers versus sisters on outcomes in children and grandchildren (all p>0.14 for difference), an unsurprising result given also no evidence of a difference among the cohort members themselves. Similarly, there was no evidence that number of older versus younger siblings was associated with descendants’ income (both p>0.55 for difference).

**Figure 4:**
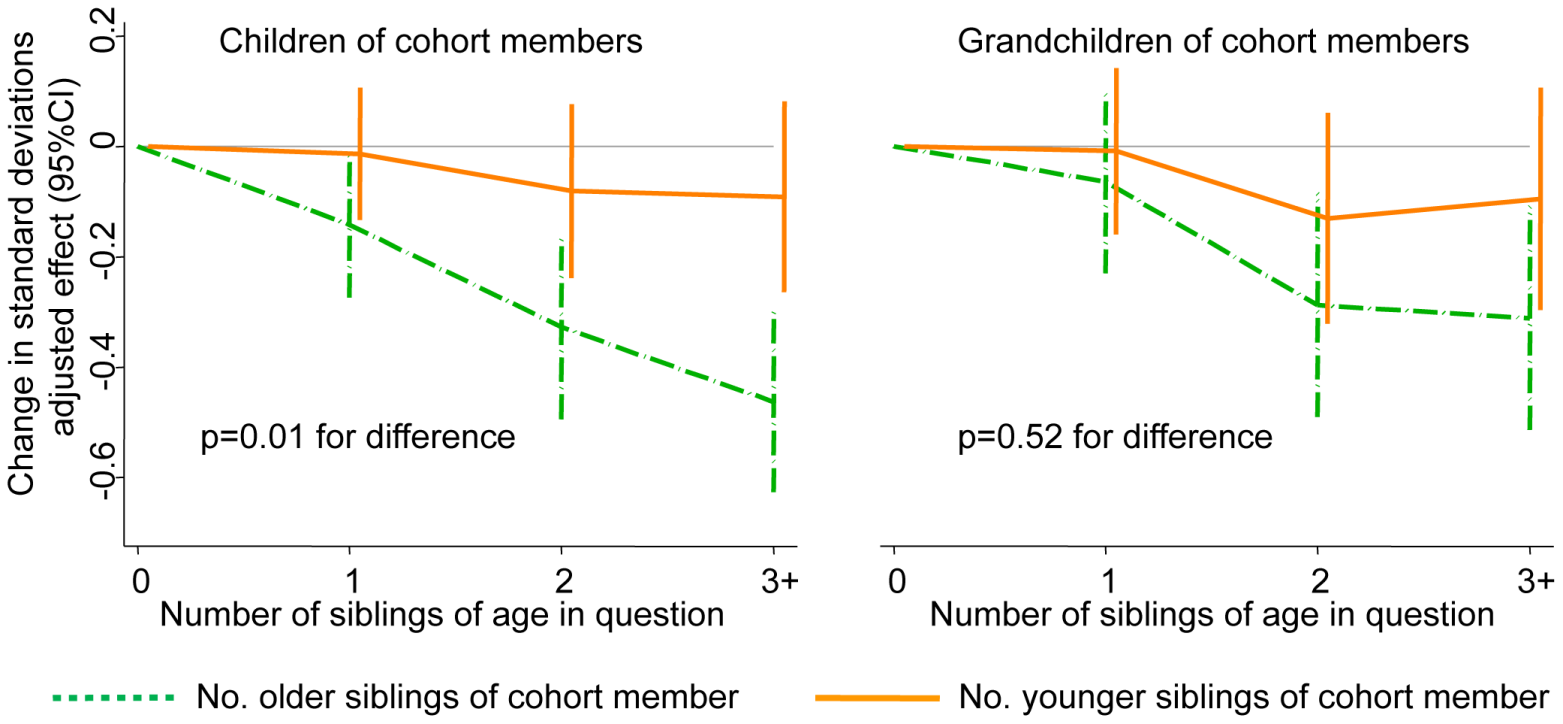
Association of the cohort members’ number of older versus younger siblings with the probability of entering university among their children and grandchildren. p-values presented are for the difference in the effect of the cohort members’ number of older versus younger siblings, from multivariable models adjusting for both sibling variables plus the other early-life characteristics shown in Table 1.

By contrast, a greater detrimental effect of older versus younger siblings was observed with respect to tertiary education in the child generation (p<0.001, see Figure 4). By the grandchild generation this difference had become non-significant, although a trend remained in the same direction (see Figure 4). Schoolmark data was not available for the child generation, and by the grandchild generation there was no evidence of a greater detrimental effect of older versus younger siblings (p = 0.76 for difference).

## Discussion

This study demonstrates that sibling configuration, rather than simply sibling number, predicts individual and descendant socioeconomic outcomes in a modern population born in early twentieth century Sweden. Specifically, we find that individuals growing up with several older siblings achieve lower schoolmarks and are less likely to progress to tertiary education, when compared to those that grow up with an equal number of younger siblings. Such educational effects are also carried forward to future generations, with a higher number of older vs. younger siblings also predicting lower educational attainment in the cohort member’s children. We found no evidence, however, that adult income is influenced by the relative age of siblings, and the sex of siblings was also not associated with any indicator of socioeconomic success.

Our findings with respect to sibling age and educational outcomes are consistent with previous studies demonstrating that later-born children in modern post-industrial low fertility societies are disadvantaged in terms of IQ, educational achievement and educational attainment [2], [3], [5], [45], [50]. They also extend our previous research into the long-term consequences of family size on descendant socioeconomic success [20], by identifying that the multigenerational effects of high fertility upon education outcomes are most strongly driven by the disadvantages of being a later-born child. These within-family inequalities in child and descendant outcomes receive comparatively little attention from academics and policy-makers, but may be comparable in magnitude to the between-family inequalities driven by factors such as parental social class [50].

Interestingly, however, the relative age of siblings did not influence adult income in either the cohort member or subsequent generations. This result is surprising since schoolmarks and educational attainment were at least somewhat predictive of adult income (observed Pearson correlations 0.08–0.29). Thus our findings caution that disadvantages in early life cognitive development and education associated with family structure cannot necessarily be extrapolated to later adult socioeconomic outcomes. A number of factors may explain why those with older vs. younger siblings achieve similar levels of adult income despite their demonstrated disadvantage in terms of education. Income is less strongly associated with total sibling number than the two educational outcomes (Figure 1; see also [20]), plausibly because the dilution of parental resources is a weaker determinant of offspring outcomes during later adult life than during childhood and the transition to adulthood. As such, one possibility is simply that this study lacked power to detect relatively small differential effects of sibling age on income.

Another possible explanation is that having older siblings may confer alternative advantages which are not measured by this study. For example, as noted in the introduction, British children with several older as opposed to several younger siblings have been found to score significantly better on validated, parent-rated measures of child mental health [47]. This finding requires wider replication, but poor child mental health is associated with a range of adverse outcomes in later life [48], and thus could have knock-on negative influences on adult earning potential. It is unclear why those with older siblings in particular should experience a lower incidence of child mental health problems, but several studies have suggested that sibling relationships can be an important source of social and emotional support. For example Grass, Jenkins & Dunn [54] found that self-reported affectionate relationships between siblings had a protective effect on adjustment to stressful life events. Downey & Condron [55] also report that children with siblings score higher on measures of interpersonal skills, suggesting that growing up in a multiple child family may enhance future abilities to navigate social relationships (but see [56]). More research is required on the impact of sibling configuration on such alternative measures of child development and adult outcomes.

We find that the sex of siblings exerted no influence on the socioeconomic success of cohort members or their descendants. This finding is consistent with previous reports that parental investment in modern populations like contemporary Sweden shows at most only a slight sex preference [36], and so growing up with relatively more brothers vs. sisters has no discernable impact on indicators of socioeconomic outcomes (see also [40]). We also found no evidence that the number of brothers vs. sisters interacted with the sex of the child, contradicting the expectation of some scholars that sibling competition will be most pronounced when siblings are of the same sex (e.g. [57]).

Our study has a number of notable methodological advantages. Firstly, we were able to consider simultaneously three important socioeconomic indicators (school achievement, educational attainment and income) measured at different points of the life course. Second, we considered these measures both for the cohort member and for their children and grandchildren. Finally, we were able to adjust our analyses for birth characteristics and the socioeconomic position of parents, which may otherwise confound the effects of resource dilution. As we demonstrate, the differential consequences of growing up with several older vs. younger siblings only became apparent after making statistical adjustments for these factors – in particular for the fact that the low birth order children are most likely to face the disadvantages of being born to younger and unmarried mothers. Future studies should be aware of these potentially confounding relationships, particularly in the context of modern populations where childbirth is relatively more common outside of marriage.

One important limitation of our study is that information on half siblings was only collected at 1930 and after this period we only have information on full siblings. These data restrictions mean that we were unable to fully characterise each cohort member’s full sibling experience in early life, and this is likely to have introduced some measurement error. Another limitation is that our measure of adult family income only captures wealth creation, and excludes wealth ownership, which may be more strongly influenced by patterns of inheritance [21]. However, we anticipate that inheritance sums are usually bequeathed equally to children in modern post-industrial populations and so we do not suspect this pathway will influence relationships between birth order and wealth ownership. It is also important to note that although the results presented here are consistent with popular hypothesised mechanisms of family resource dilution [4], they do not demonstrate causality. Several studies have now shown that children growing up with many siblings, and with older siblings in particular, spend less time with their parents engaged in care activities [12], [13], [41]. Few such studies, however, have also directly tested whether such associations mediate the lower levels of educational achievement and attainment (see [13] for a notable exception). Understanding these mechanisms more fully may ultimately inform the design of interventions that mitigate not only between-family inequalities generated by family size but also the within-family inequalities generated through sibling configuration.

## Supporting Information

File S1 This file provides further information on (a) how we derived sibling configuration data for each cohort member from multiple sources and (b) an assessment of the potential for measurement error by sibling sex and age.
(DOC)
