# Supplementary material for: Sibling Configuration Predicts Individual and Descendant Socioeconomic Success in a Modern Post-Industrial Society
Source: PLoS One. 2013 Sep 6;8(9):e73698. doi: 10.1371/journal.pone.0073698 (PMC3765256; doi:10.1371/journal.pone.0073698)
Supplement: File S1 — This file provides further information on (a) how we derived sibling configuration data for each cohort member from multiple sources and (b) an assessment of the potential for measurement error by sibling sex and age. (DOC) [file pone.0073698.s001.doc]

**Sibling configuration predicts individual and descendant socioeconomic success in a modern post-industrial society.**

**David W. Lawson** ***1**

**Arijeta Makoli 2**

**Anna Goodman 2,3**

1 - Department of Anthropology, University College London, London, UK

2 - Centre for Health Equity Studies (CHESS), Stockholm University/Karolinska Institute, Stockholm, Sweden

3 - Faculty of Epidemiology and Population Health, London School of Hygiene & Tropical Medicine, London, UK

* - Corresponding author. Email: d.lawson@ucl.ac.uk

**Supplementary Information S1**

**Tracing cohort members to the 1930 census and extracting sibling data**

From previous archive data collection, we knew the parish that children lived in at birth and at age 10. We used this to trace children to the 1930 census, searching census records from each parish for a child with the right name and right year of birth. We then verified the children were correctly identified using the name and birthyear of the parents. The census collected information on all individuals living within a family household, identified based on sharing a living space. The father was typically listed as the head of the family and his relationship to all others in the family was stated. Examples of descriptions used to summarise this relationship in the census include ‘son’, ‘adopted son’ or ‘wife’s son from a previous marriage’. We used this information to assign each child in the household a sibling type relative to our cohort member. We also used the information given on the sex and birth year of each sibling to determine whether they were brothers or sisters and whether they were older or younger than the cohort member.

**Estimation of cohort members’ number of siblings of different types**

Table 1: Triangulation of obstetric and census data to characterise sibling characteristics

| **No. older siblings**† | Highest of:  1) recorded number of previous live births in the obstetric data  2) no. older siblings observed in the 1930 census  Pearson correlation 0.86 between obstetric and census data |
| --- | --- |
| **No. younger siblings**† | Highest of:  1) observed number of subsequent live births in the obstetric data  2) no. younger siblings observed in the 1930 census  Pearson correlation 0.75 between obstetric and census data |
| **No. brothers** | Sum of :  * no. older brothers, estimated as the highest of:  1a) [if born 1924-1929]: No. recorded in obstetric records  1b) [if born 1915-1923]: No. observed in obstetric records IF total number of observed brothers + older sisters was equal to recorded number of previous live births (i.e. no missing older siblings). Otherwise this individual was excluded for this outcome on the basis that we did not have reliable data on the sex of their older siblings.  2) No. observed in 1930 census.  plus  * no. younger brothers, estimated as the highest of:  1) No. observed in obstetric records  2) No. observed in 1930 census.  plus no. twin/triplet brothers observed in obstetric records  Pearson correlation 0.70 between obstetric and census data |
| **No. sisters** | Estimated as for no. brothers.  Pearson correlation 0.71 between obstetric and census data |

†We did not count twins as either older or younger siblings, but used obstetric information to create a separate variable to capture twin/triplet status.

**Differential measurement error by sibling sex and age**

If we measured one sibling type more accurately than another, then this could bias our findings by leading us to underestimate the effect of the poorly-measured sibling type. With respect to sibling sex, this did not seem likely to be an issue. As shown in Figure 5, the proportion of female siblings was very similar to the expected value of 49% in older and younger siblings of almost all ages. The only exception was that the proportion of females dropped to 40-45% among those born more than 13 years before the cohort member, probably reflecting older sisters getting married and leaving home earlier than older brothers. Only 5% of siblings were aged more than 13 years more than our cohort members, however, suggesting that this will not have introduced any important differential measurement error.

Figure 5: Percent females among siblings identified in the 1930 census for cohort members born 1915-1924, according to the age gap between the sibling in question and the cohort member.


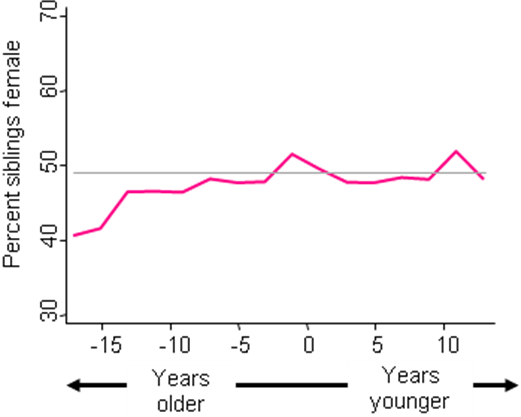


As judged by the distribution of birth years of older and younger siblings, by 1930 almost all younger siblings that would ever be born had already been born to cohort members born in 1915. For those born 1919 most siblings had been born, whereas for those born 1924 (the youngest cohort members included in our sample) the fraction was somewhat under half (see Figure 6). Our substantive findings were, however, almost identical in analyses restricted to those born 1915-1919: indeed, for schoolmarks there was if anything an even greater difference between older and younger siblings (see Figure 7). As such, it did not seem that the observed differential effect of sibling age could be explained by greater measurement error with respect to younger siblings.

Figure 6: Distribution of birth years of siblings identified in the 1930 census, for cohort members born in 1915, 1919 and 1924


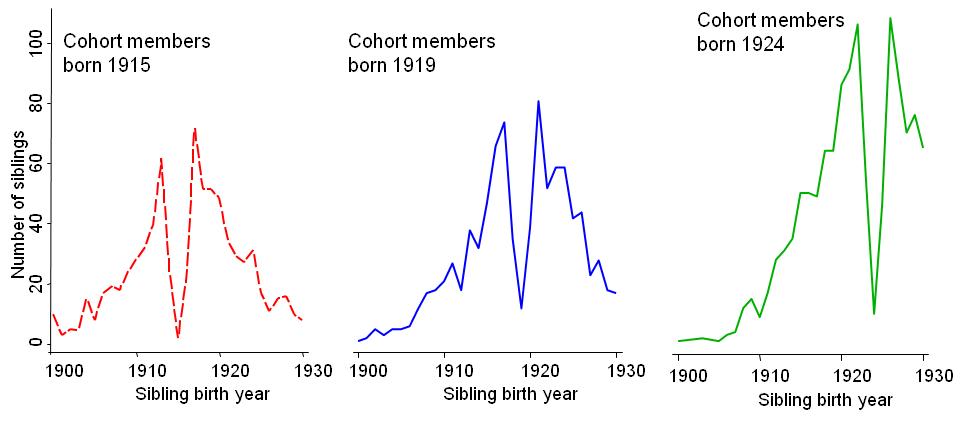


Figure 7: Comparison of the effects of sibling age upon educational outcomes in the full study population (born 1915-1924) versus the subpopulation born 1915-1919


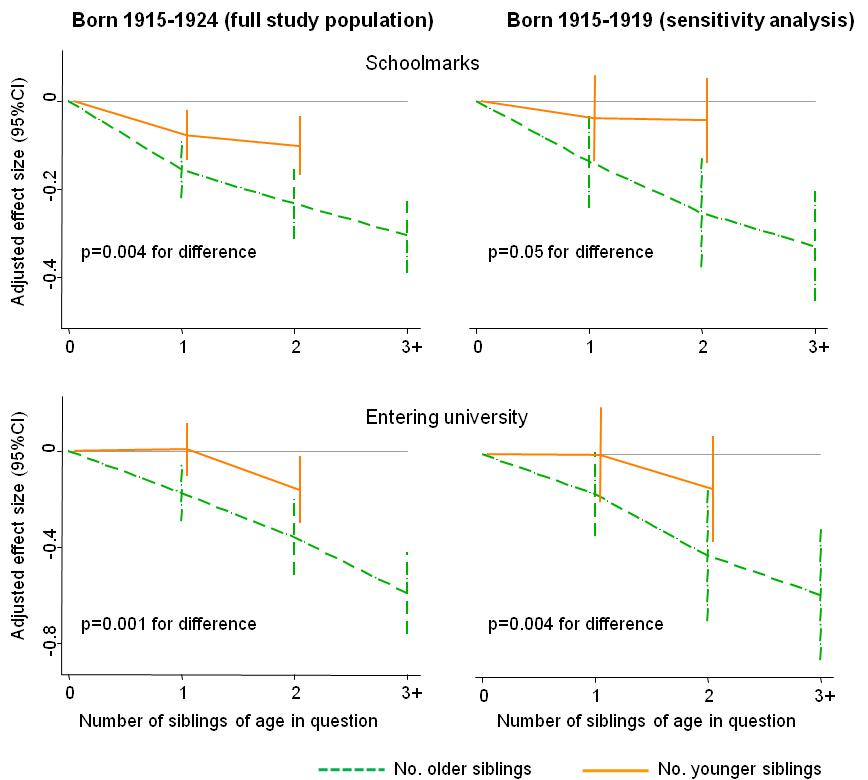


Younger siblings grouped at 2+ not at 3+ because of small numbers in the 1915-1919 subsample
